# The TTF-1 and Napsin A Trap: Metastatic Endometrial Carcinoma Masquerading as Lung Primary

**DOI:** 10.1177/10668969251403167

**Published:** 2025-12-11

**Authors:** Carmen Alfonso-Rosa, Jesús Machuca-Aguado, Ana María Montaña-Ramírez, Francisco Javier Rubio-Garrido

**Affiliations:** 1 Pathology Department, 16582 Virgen Macarena University Hospital, Seville, Spain

**Keywords:** immunohistochemistry, endometrial carcinoma, pitfalls, lung cancer

The detection of a pulmonary lesion suspicious for malignancy always raises the differential diagnosis between primary lung neoplasia and extrapulmonary metastasis. Immunohistochemistry (IHC) has facilitated the categorization of such lesions and often allows more precise distinction between primary and metastatic tumors. However, limitations in routine IHC use must be acknowledged.

We present a 79-year-old woman diagnosed 7 years prior at another institution with low-grade endometrioid endometrial adenocarcinoma (Grade 2), Stage III. The patient responded well to surgical treatment with adjuvant chemoradiotherapy. During follow-up 3 years later, a radiologically suspicious nodule in the right lower lobe was identified. Histologically, it corresponded to an adenocarcinoma with an immunohistochemical profile of KRT7+/KRT20-/TTF-1+/Napsin A+. This lesion was initially diagnosed as a well-differentiated acinar lung adenocarcinoma (Stage IB) and treated surgically without adjuvant therapy. The pathologist who made this initial diagnosis was aware of the patient's prior history of endometrial carcinoma; however, PAX8 immunostaining was not available at that time in the laboratory. Next-generation sequencing (NGS) revealed mutations in *KRAS, PIK3CA*, and *ARID1A*, with no copy number variations.

Surveillance imaging identified a new suspicious nodule in the left lower lobe, hypermetabolic dorsal lesions, and a left supraclavicular lymph node. A core needle biopsy of the pulmonary nodule was performed. Histology showed a glandular proliferation with cribriform architecture, elongated nuclei, eosinophilic cytoplasm, increased nuclear-to-cytoplasmic ratio, loss of cellular polarity, and frequent mitotic figures. IHC revealed diffuse positivity for TTF-1, PAX8, estrogen receptors, progesterone receptors, and Napsin A, with negativity for GATA3. DNA mismatch repair proteins were retained, and p53 exhibited a wild-type pattern.

Review of prior specimens (hysterectomy, bronchoscopy, and lobectomy) confirmed identical IHC profiles and mutational patterns via NGS. Despite TTF-1 and Napsin A expression, both pulmonary lesions were concluded to represent metastatic endometrial carcinoma.

**Figure 1.**
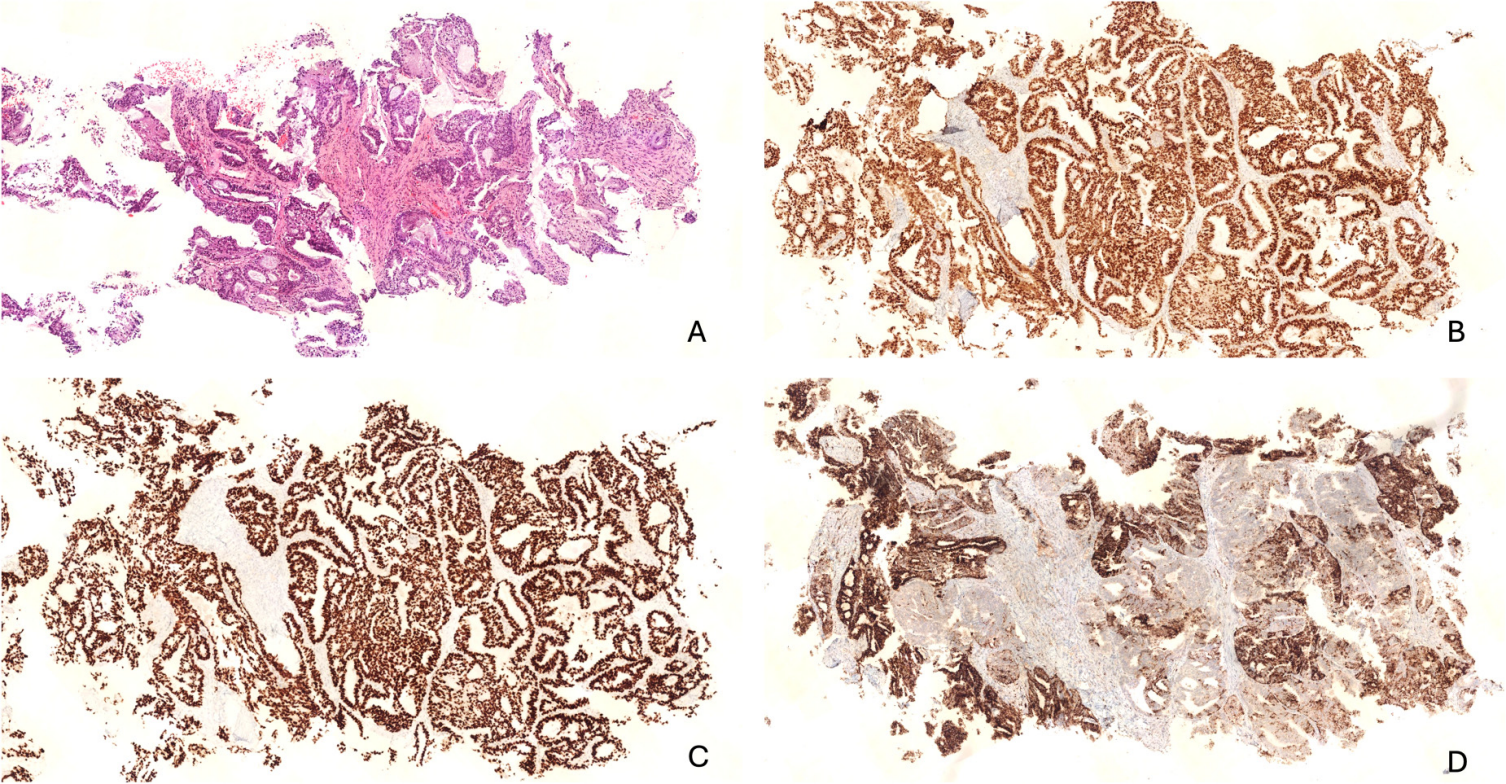
Lung biopsy showing a metastatic adenocarcinoma. (A) H&E stain highlights round to oval glandular units with central lumina. (B) Immunohistochemistry for PAX8 shows strong nuclear positivity, supporting a Müllerian origin. (C) TTF-1 and (D) Napsin A show intense nuclear and cytoplasmic staining, respectively.

**Figure 2.**
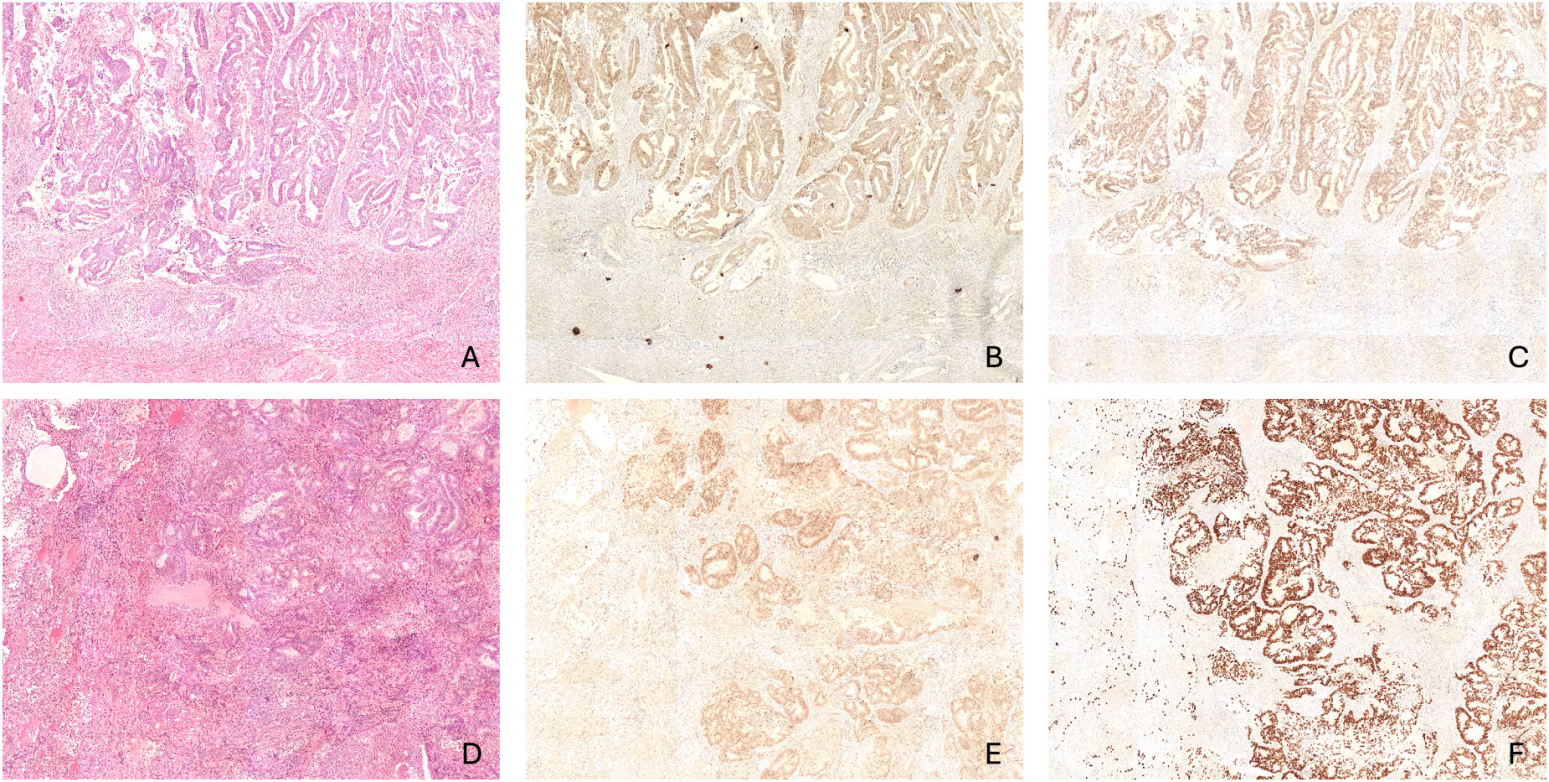
Primary uterine grade 2 endometrioid adenocarcinoma. (A) Primary uterine tumor showing characteristic morpholog, showing (B) diffuse PAX8 positivity and (C) diffuse TTF-1 positivity. (D) The subsequent pulmonary metastasis in the lobectomy specimen also demonstrated (E) diffuse positivity for PAX8 (F) and TTF-1.

Figures 1 and 2 illustrate the common morphological and immunohistochemical features. Figure 1 corresponds to the lung biopsy, while Figure 2 shows the hysterectomy and lobectomy specimens.

The situation described here represents a paradigmatic example of how isolated interpretation of immunohistochemical markers can lead to diagnostic errors. The original diagnosis of the first pulmonary lesion as a primary lung adenocarcinoma relied heavily on TTF-1 and/or Napsin A positivity, without adequately considering clinical history, morphological features, or complementary markers that could have suggested metastasis from endometrial carcinoma.

Firstly, TTF-1, while a valuable marker for pulmonary adenocarcinoma, lacks absolute specificity. Over the years, its expression has been documented in nonpulmonary tissues, both neoplastic and nonneoplastic.^1–4^ Siami et al^2^ demonstrated TTF-1 positivity in 19% (6/32) of endometrioid endometrial adenocarcinomas, though only 2 neoplasms (1 low-grade and 1 high-grade) showed diffuse immunoreactivity. Ervine et al^3^ later analyzed 319 endometrial carcinomas and found focal TTF-1 positivity in 7% (2/29) of clear cell carcinomas and 9% (8/89) of high-grade serous carcinomas. Among endometrioid adenocarcinomas, diffuse and focal TTF-1 expression was observed in 7% and 4% of high-grade carcinomas, respectively. Notably, only 1% (1/100) of low-grade endometrioid carcinomas exhibited diffuse positivity.

Mills et al^4^ expanded this evidence in a study of 300 endometrioid carcinomas, identifying TTF-1 positivity in 3% (9/300) of cases, with rates dropping to 1.25% (3/240) in pure endometrioid subtypes. Only one patient with a low-grade endometrioid carcinoma with mucinous differentiation, hormone receptor expression, and *KRAS/ARID1A/PIK3CA* mutations-showed intense, diffuse staining, mirroring our patient's molecular profile. More recently, Möller et al^1^ evaluated TTF-1 expression in over 152 neoplasms using tissue microarrays, reporting positivity in 4.2% (12/295) of endometrioid endometrial carcinomas, with only 4 lesions showing strong reactivity.

Napsin A is a widely used immunohistochemical marker in the diagnosis of pulmonary adenocarcinomas, with high reported sensitivity and specificity—particularly when interpreted in conjunction with TTF-1. Different studies have shown that coexpression of Napsin A and TTF-1 is highly suggestive of a primary pulmonary origin, being present in up to 91% of primary lung adenocarcinomas, while this pattern is exceptionally rare in metastatic tumors of nonpulmonary origin, including those from the gynaecological tract.^5,6^ In endometrial adenocarcinomas, Napsin A expression varies by histological subtype: it is relatively frequent in clear cell carcinomas (up to 66%), less common in serous carcinomas (9%), and rare in endometrioid subtypes (approximately 4.5-6.5%), where it typically exhibits only focal staining.^5^ Importantly, no endometrioid adenocarcinomas have been documented to coexpress with a diffuse pattern both TTF-1 and Napsin A. While Napsin A positivity may initially suggest a pulmonary origin, its expression is not exclusive and must be interpreted within the appropriate clinical context.

Secondly, a comparative morphological analysis of the pulmonary lesions and the original endometrial tumor could have revealed striking architectural and cytological similarities. Such overlap underscores the importance of revisiting prior specimens when evaluating new lesions in patients with a cancer history.

Finally, the incorporation of additional markers, such as PAX8 and hormone receptors, would have challenged the assumption of pulmonary origin despite TTF-1 and/or NapsinA positivity. PAX8, a transcription factor involved in the development of the thyroid, kidney, and Müllerian system, demonstrates near 100% sensitivity for endometrial origin and is consistently negative in primary lung adenocarcinomas.^7,8^ Hormone receptors (estrogen and progesterone) expression is characteristic of endometrioid carcinomas but exceptionally rare in lung primaries.^9^

Another important differential diagnosis considered was mesonephric-like adenocarcinoma, given the well-formed glandular architecture, diffuse TTF-1 expression, absence of microsatellite instability, wild-type p53 pattern, and the presence of concurrent *KRAS*, *PIK3CA*, and *ARID1A* mutations—features that, alongside the aggressive clinical course, could initially raise this possibility. However, classic mesonephric-like adenocarcinomas typically display an infiltrative growth pattern with angulated tubules, glandular complexity, luminal eosinophilic secretions, and an immunophenotype characterized by negativity for estrogen and progesterone receptors and positivity for mesonephric markers such as GATA3 and CD10 (with luminal staining).^4,10^ In our patient, diffuse ER and PR positivity combined with the absence of GATA3 and luminal CD10 staining is inconsistent with a mesonephric profile and supports a diagnosis of endometrioid origin. Furthermore, the presence of *ARID1A* mutations—more characteristic of endometrioid carcinomas than of mesonephric-like tumors—reinforces this interpretation. Taken together, the morphological, immunohistochemical, and molecular findings strongly support a diagnosis of metastatic endometrioid carcinoma over mesonephric-like adenocarcinoma.

To conclude, this report highlights the risks of overrelying on isolated immunohistochemical markers like TTF-1 or NapsinA. While we do not dispute the utility of IHC, we emphasize the need for a holistic approach that integrates morphological analysis, broad IHC panels and clinical history. In patients with gynecological cancer histories, TTF-1/NapsinA expression shouldńt be automatically interpreted as suggestive of a second lung adenocarcinoma and PAX8 should be routinely included, as it is highly sensitive for endometrial origin and consistently negative in primary lung adenocarcinomas. This approach can help prevent misdiagnosis when TTF-1 and NapsinA are positive.
